# Supplementary material for: Salacia chinensis L. Stem Extract Exerts Antifibrotic Effects on Human Hepatic Stellate Cells through the Inhibition of the TGF-β1-Induced SMAD2/3 Signaling Pathway
Source: Int J Mol Sci. 2019 Dec 13;20(24):6314. doi: 10.3390/ijms20246314 (PMC6940887; doi:10.3390/ijms20246314)
Supplement: Supplementary file 1 [file ijms-20-06314-s001.pdf]

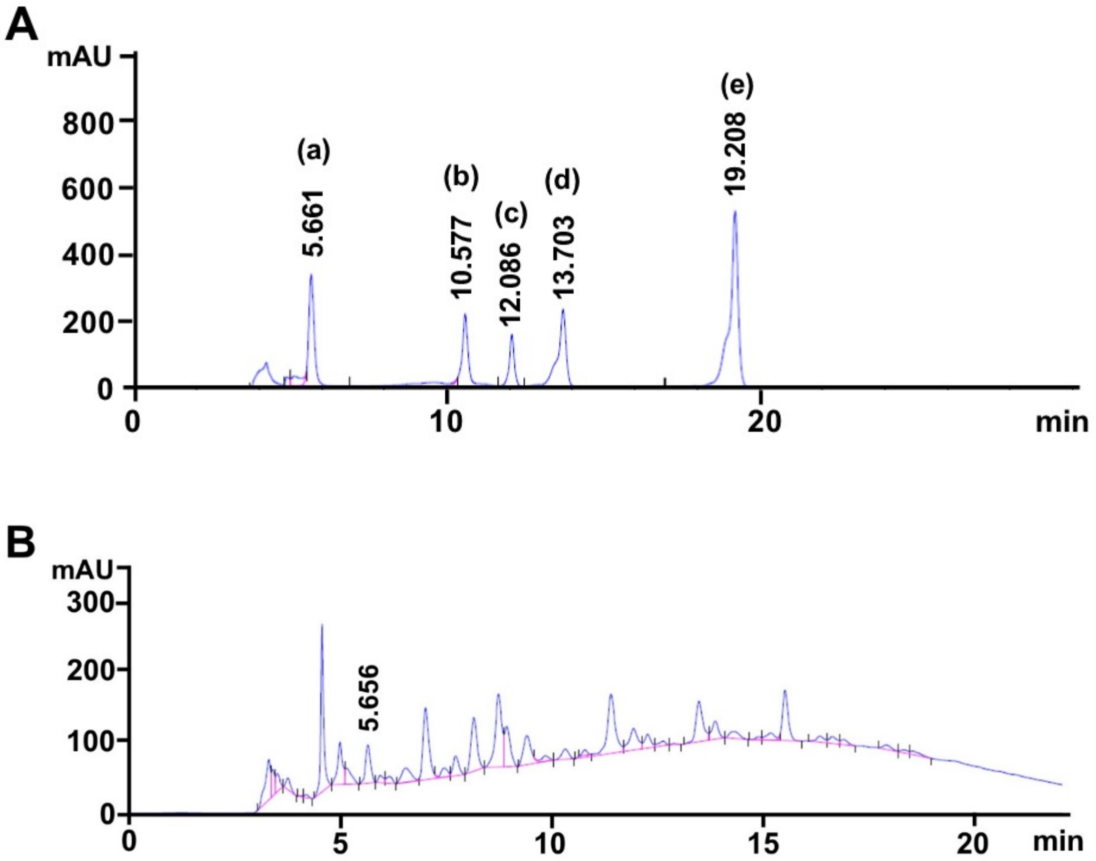

Supplementary Figure 1. HPLC chromatogram of standard compounds (**A**); (a) gallic acid, (b) chlorogenic acid, (c) mangiferin, (d) vanillic acid, (e) ferulic acid, and *Salacia chinensis* L. (SC) stem extract (**B**).
